# Effects of Lipid Peroxidation-Mediated Ferroptosis on Severe Acute Pancreatitis-Induced Intestinal Barrier Injury and Bacterial Translocation

**DOI:** 10.1155/2021/6644576

**Published:** 2021-06-22

**Authors:** Deliang Ma, Pengling Jiang, Yingjian Jiang, Hongbo Li, Dianliang Zhang

**Affiliations:** ^1^Center of Colon and Rectum, Qingdao Municipal Hospital, Qingdao University, No. 1 Jiaozhou Road, Qingdao, 266011 Shandong, China; ^2^Breast Surgery, Qingdao Municipal Hospital, Qingdao University, No. 1 Jiaozhou Road, Qingdao, 266011 Shandong, China

## Abstract

Ferroptosis is a recently recognized type of regulated cell death characterized by iron- and lipid peroxidation-mediated nonapoptotic cell death. However, whether ferroptosis is involved in severe acute pancreatitis- (SAP-) induced intestinal barrier injury is unknown. The aim of this study was to investigate whether ferroptosis is involved in SAP-induced intestinal barrier injury, particularly intestinal epithelial cell (IEC) death, and determine whether the inhibition of ferroptosis would ameliorate intestinal barrier injury and prevent bacterial translocation (BT). Sodium taurocholate (5%) was retrogradely perfused into the biliopancreatic duct to establish a rat model of SAP. The rats were divided into three groups: sham operation (SO), SAP-induced intestinal barrier injury (SAP), and ferroptosis inhibitor liproxstatin-1 (SAP + Lip). Serum indexes were measured in the rats. In addition, the biochemical and morphological changes associated with ferroptosis were observed, including iron accumulation in intestinal tissue, lipid peroxidation levels, and mitochondrial shrinkage. Hematoxylin staining and eosin staining were used to assess histological tissue changes. Western blot, RT-PCR, and immunofluorescent staining were performed to analyze the expression of ferroptosis-related proteins and genes as well as tight junction. BT was detected by 16S rDNA sequencing analysis. The results indicated that ferroptosis was significantly induced in the IECs from rats with SAP and ferroptosis was mediated by lipid peroxidation. The specific lipid peroxidation of IECs clearly upregulated ferroptosis and exacerbated intestinal barrier injury. Furthermore, treatment with liproxstatin-1 lowered the levels of serum damage markers, decreased lipid peroxidation, and alleviated intestinal and acute remote organ injury in SAP rats. In addition, inhibition of ferroptosis reduced BT. Our findings are the first to demonstrate that ferroptosis contributes to SAP-induced intestinal barrier injury via lipid peroxidation-mediated IEC death. These results suggest that ferroptosis is a potential therapeutic target for SAP-induced intestinal barrier injury.

## 1. Introduction

Acute pancreatitis is a common acute abdominal condition with rapid onset, and it can lead to high mortality when it develops into severe acute pancreatitis (SAP) [1]. Secondary infection of the pancreas is considered to be the main cause of the high mortality of SAP [2]. The intestinal mucosa is a major anatomical and functional barrier, which prevents potentially harmful malignant intestinal bacteria and endotoxins from entering the systemic circulation and extraintestinal tissues [3]. The relationship between intestinal barrier dysfunction and SAP has been confirmed. SAP can destroy normal intestinal mucosal function, leading to bacterial and endotoxin translocation into systemic circulation and eventually leading to secondary infection, systemic inflammatory response syndrome, and multiple organ dysfunction syndrome [4, 5]. Therefore, it is of great significance to explore effective therapies to protect the intestinal barrier function and prevent gut-derived infection caused by SAP.

The mechanisms of intestinal mucosal barrier dysfunction induced by SAP have not been fully elucidated. The activation of inflammatory responses and oxidative stress is considered to play important roles in the intestinal injury of SAP [6]. However, the destruction of intestinal integrity is believed to be the main mechanism of endotoxin/bacterial translocation (E/BT), which may lead to the most serious complications in the late stage of SAP, including sepsis [7]. Our previous work showed that changes in the protein composition of paracellular tight junctions (TJ) are the major factor driving SAP to increase intestinal permeability [8]. In addition, a previous study has found that inhibiting apoptosis of intestinal epithelial cells (IECs) can prevent the destruction of intestinal integrity and reduce bacterial translocation [9]. The massive death of IECs is an important cause of intestinal mucosal barrier dysfunction, which will lead to systemic inflammation and remote organ dysfunction [10]. However, in addition to apoptosis, SAP-induced intestinal barrier damage may also involve other types of cell death. Therefore, we speculated that ferroptosis of IECs may be a possible mechanism leading to the increase of intestinal permeability and subsequent E/BT.

Ferroptosis, a newly discovered regulatory cell death, is an iron-dependent and caspase-independent nonapoptotic form of cell death, which differs from other types of cell death in terms of biochemistry, cell morphology, and genetics [11, 12]. The main morphological features of ferroptosis are the narrowing of mitochondria, the thickening of mitochondrial membranes, and the decrease of mitochondrial crista [12]. Iron metabolism and lipid peroxidation are considered to be the key mediators of ferroptosis. The biochemical mechanism of ferroptosis is the formation of lipid radicals catalyzed by iron, combined with the depletion of glutathione (GSH) or the inactivation of lipid repair enzyme GSH peroxidase 4 (GPX4) [13, 14]. Additionally, lipid peroxidation is affected by several lipids and enzymes. Therefore, the oxidation of polyunsaturated fatty acids was derived from the catalytic pathways of acyl-CoA synthetase long chain family member 4 (ACSL4) and lysophosphatidylcholine acyltransferase 3 (LPCAT3), which mediate lipotoxicity in ferroptosis [15, 16]. Numerous studies have shown that ferroptosis participates in a variety of pathological processes, including kidney, brain, heart, and liver diseases [17]. Lipophilic radical traps, including ferrostatin-1 (Fer-1), liproxstatin-1 (Lip-1), and vitamin E, can neutralize the deleterious effects of lipid peroxidation during the execution of ferroptosis. Nevertheless, the role of ferroptosis in SAP-induced intestinal barrier injury has not been investigated.

In the present study, the effects and mechanisms of ferroptosis in SAP-induced intestinal barrier injury were investigated. We report for the first time that ferroptosis is involved in the IEC death of SAP-induced intestinal mucosal barrier dysfunction, which is mediated by lipid peroxidation. Our results show that the inhibition of ferroptosis is an important mechanism for protecting intestinal barrier injury in SAP rats, suggesting that ferroptosis is a potential therapeutic target for SAP treatment.

## 2. Materials and Methods

### 2.1. Animals and Model

This study was approved by the Animal Care and Experiment Committee of Qingdao University (Qingdao, China) and carried out in accordance with the National Institutes of Health guidelines for laboratory animals. Ninety adult male SPF Sprague-Dawley rats, 8 weeks old, 200–250 g, were provided by the animal center of Qingdao University. The rats were randomly divided into three groups: sham operation group (SO), SAP group (SAP), and SAP plus Lip-1 treatment (SAP + Lip) group. The rats in three groups were further divided into 6, 12, and 24 h time groups (10 rats in each subgroup).

The rats fasted 12 h before the experiments. After anesthesia with 3% sodium pentobarbital (20 mg/kg), freshly prepared 5% sodium taurocholate (Sigma) solution (1 ml/kg) was injected into the bile-pancreatic duct under standard pressure-controlled infusion (SK-500II microinfusion pump, 0.02 ml/min) to induce the SAP model [18]. In the SO group, the same amount of sterile saline was injected into the duct. The SAP + Lip group received intraperitoneal injection of the ferroptosis inhibitor Lip-1 (Selleck), at the concentration of 10 mg/kg 1 h before the establishment of the SAP model, following the previous study protocol [19]. Meanwhile, in the SO and SAP groups, the rats were injected intraperitoneally with the same volume of vehicle.

### 2.2. Sample Collection

After inducing pancreatitis, the rats were anesthetized again at designated time points (6, 12, and 24 h). Blood samples were collected from the inferior vena cava and divided into two microtubes. One blood sample was centrifuged at 3000 rpm at 4°C for 5 min, and the supernatant was stored at −20°C for subsequent serum analysis. The other sample of blood was collected in EDTA microtubes and stored at −20°C for bacterial DNA detection. The head of the pancreas, ileal tissue adjacent to the cecum, lung, and kidney tissues were removed and fixed with 4% paraformaldehyde for sectioning, while other tissues were stored at −80°C for later analysis.

### 2.3. Serum Assays

The activities of serum amylase (AMY) and lipase (LIPA) in blood samples were measured by an automatic biochemical analyzer (Olympus, Tokyo, Japan). Serum tumor necrosis factor- (TNF-) *α*, interleukin- (IL-) 6, creatinine (Cr), and blood urea nitrogen (BUN) were detected according to the manufacturer's instructions using a standard diagnostic kit (Jiancheng Biotech, Nanjing, China). Diamine oxidase (DAO) was measured using the rat DAO ELISA kit (Jiancheng Biotech, Nanjing, China). The concentration of serum endotoxin was detected with the EKT-5 M set dynamic Gram-negative bacteria test kit (Jin Shanchuan, Beijing, China).

### 2.4. Iron Measurements

Fresh ileum was homogenized in phosphate-buffered saline (PBS) immediately. After centrifugation, the supernatant was collected and the iron content was determined with an iron analysis kit (Abcam) according to the manufacturer's instructions.

### 2.5. Malondialdehyde (MDA), GSH, and GPX4 Determination

In order to determine the level of lipid peroxidation in ileum at different time points after SAP, the levels of malondialdehyde (MDA), GSH, and GPX4 activity were measured using commercially available kits (Jiancheng Biotech, Nanjing, China).

### 2.6. Transmission Electron Microscopy (TEM)

Fresh ileal tissue was washed with precooled PBS (pH 7.4) and then postfixed in 2.5% phosphate-buffered glutaraldehyde and 1% osmium tetroxide. The ileal tissue samples were sectioned using an ultramicrotome and stained with uranyl acetate and lead citrate. The ultrastructural images of IECs were then captured by TEM (Hitachi HT7700, Japan).

### 2.7. Histopathological Evaluation and Immunofluorescence

The tissues of the pancreas, ileum, lung, and kidney were fixed with 4% formalin and embedded in paraffin for histological analysis. Tissue sections (5 *μ*m) were stained with hematoxylin and eosin (H&E), and the morphological changes were observed with a light microscope. The degrees of injury of the pancreas, ileum, lung, and kidney were scored based on previous criteria [20–23]. Histopathological changes of the pancreas were analyzed according to the Schmidt score, including the degree of edema, the number of leukocyte infiltration, hemorrhage, and necrosis lesions, with a maximum score of 16. The ileum histopathology changes were examined based on Chiu's score with a maximum score of 5. The scoring criteria in detail are as follows: (1) grade 0, normal mucosal villi; (2) grade 1, development of congestion; (3) grade 2, extension of the subepithelial space with moderate lifting of the epithelial layer from the lamina propria; (4) grade 3, massive epithelial lifting down the sides of villi; (5) grade 4, denuded villi with lamina propria and dilated capillaries exposed; and (6) grade 5, digestion and disintegration of lamina propria, hemorrhage, and ulceration.

The expression of ACSL4 and TJ proteins in rat IECs was determined by immunofluorescence double staining. The immunostaining of the tissue was performed as described earlier [24]. OTC-embedded sections (5 *μ*m) were dried at 37°C in an oven. For immunostaining, the slides were cultured overnight with the following primary antibodies: rabbit anti-ACSL4 (Abcam), rabbit anti-ZO-1 (Abcam), rabbit anti-occludin (Zymed Laboratories), and rabbit anti-claudin-2 (Zymed Laboratories). After rinsing with PBS, Alexa Fluor goat anti-rabbit IgG was added. The sections were incubated at room temperature for 30 min, and the nuclei were stained with 4′,6-diamidino-2-phenylindole (DAPI) (Life Technologies) for 5 min. Confocal images of the ileal sections were obtained using a confocal laser microscope (Leica, Wetzlar, Germany).

### 2.8. Bacterial Detection by 16S rDNA Sequencing

The hypervariable region of the 16S rRNA gene was amplified by extracting bacterial DNA from blood samples and using the isolated DNA as template [18]. Universal primers were F1169-1187-GC and R1521-1539. Negative and positive controls were repeated twice to avoid false-positive results. After 100 ng DNA was added to the reaction mixture, polymerase chain reaction (PCR) was performed by a touchdown thermocycling program. The band sizes of PCR products (~500 bp) were consistent with those of prokaryotic 16S rRNA gene-specific amplification. The forward primers used for PCR amplification were also used for sequencing of the template (about 600 bp read lengths). The advanced BLAST search was used to compare the results of 16S rRNA sequencing with those from the Ribosomal Database Project (RDP) and National Center for Biotechnology Information (NCBI) GenBank databases.

### 2.9. Western Blot

Total protein was extracted from rat ileums, and the concentration of total protein was determined by BCA Protein Assay kit (TransGen, Beijing, China). Western blotting was performed with the following primary antibodies: ACSL4 (Abcam), GPX4 (Abcam), FTH1 (Abcam), ZO-1 (Abcam), occludin (Zymed Laboratories), claudin-1 (Santa Cruz Biotechnology), and claudin-2 (Zymed Laboratories). *β*-Actin (Sigma) was used as loading control. For protein quantification, the density of Western blot bands was measured by Image-Pro Plus 6.0 software (Media Cybernetics, Rockville, MD, USA).

### 2.10. Real-Time PCR

Total RNA was isolated from intestinal tissues with TRIzol (Invitrogen, USA) and then reverse transcribed into cDNA using a reverse transcription kit (TaKaRa, Japan). With *GAPDH* as internal control, the relative mRNA expression of the ferroptosis-related genes *ACSL4* and *IREB2* was detected using a quantitative real-time PCR kit (TaKaRa, Japan). The primer sequences of the target genes were as follows: ACSL4, forward 5′-TTGTATTGCTGCCTGTCCACTTGTT-3′ and reverse 5′-ATTCTCTTTGCCATAGCGTTTTTCT-3′; IREB2, forward 5′-ATTCTGCCTTACTCAATACGGGTCC-3′ and reverse 5′-ATTGCTTTGTTTGGTTTTCCAGTCC-3′; GAPDH, forward 5′-CGTGTTCCTACCCCCAATGT-3′ and reverse 5′-TGTCATCATACTTGGCAGGTTTCT-3′.

### 2.11. Statistical Analysis

GraphPad Prism 6.0 (GraphPad Software, San Diego, CA, USA) was used for statistical analysis. Quantitative data were expressed as mean ± standard deviation (SD). The variables between groups were compared by student's *t*-test and one-way analysis of variance (ANOVA) with post hoc Tukey's tests. Categorical data were analyzed by chi-square tests. A *p* value < 0.05 was considered as statistically significant.

## 3. Results

### 3.1. Successful Establishment of the SAP-Induced Intestinal Barrier Injury Model

**Figure 1:**
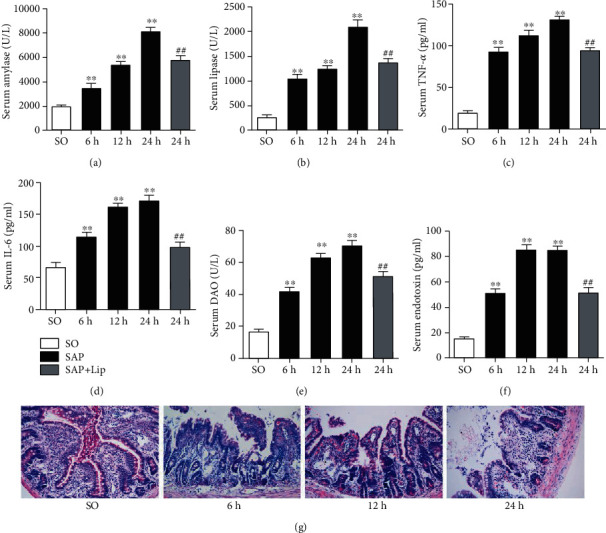
Serum biochemical indicators and intestinal histological changes at different time points. The activities of serum AMY (a) and LIPA (b) and the levels of TNF-*α* (c), IL-6 (d), DAO (e), and endotoxin (f) in the SAP group were significantly higher than those in the SO group. And the levels of AMY (a), LIPA (b), TNF-*α* (c), IL-6 (d), DAO (e), and endotoxin (f) in the SAP + Lip group at 24 h were significantly lower than those in the SAP group. Representative ileum H&E-stained sections were obtained (g) (original magnification, ×200). These values are expressed as the mean ± SD (*n* = 10). The variables between groups were compared by Student's *t*-test and one-way ANOVA. ^∗^*p* < 0.05 and ^∗∗^*p* < 0.01 vs. the SO group; ^#^*p* < 0.05 and ^##^*p* < 0.01 vs. the SAP group.

After SAP induction, all the serum injury indices increased significantly and continuously with the extension of time. As shown in Figures 1(a)–1(f), the serum activities of AMY and LIPA and the levels TNF-*α*, IL-6, DAO, and endotoxin in the SAP group were significantly increased compared to those in the SO group at each time point (*p* < 0.01). In addition, the SAP group showed representative signs of intestinal injury, such as edema, bleeding, villous exfoliation, mucosal cell necrosis, and leukocytic infiltration when compared with the SO group (Figure 1(g)). These changes showed that our SAP-induced intestinal barrier injury model was successfully established.

### 3.2. Ferroptosis Is Produced in SAP-Induced Intestinal Barrier Injury

**Figure 2:**
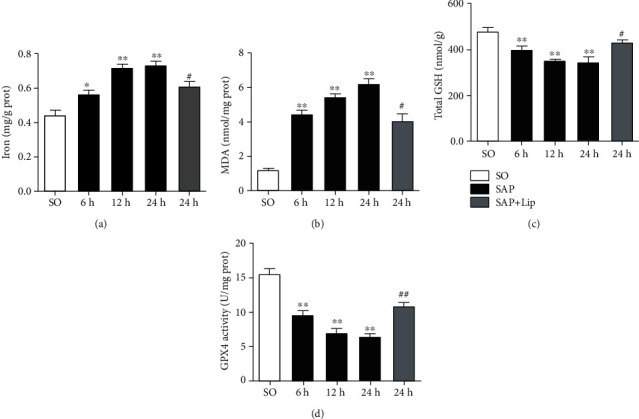
Ileum tissue was collected to detect iron content (a), MDA (b), GSH levels (c), and GPX4 activity (d) at different time points. In the SAP group, iron accumulated significantly, MDA content increased significantly, and GSH levels and GPx4 activity decreased. Lip-1 treatment decreased the iron and MDA content in intestines but markedly increased the GSH levels as well as GPX4 activity. All results are expressed as the mean ± SD (*n* = 10). The variables between groups were compared by Student's *t*-test and one-way ANOVA. ^∗^*p* < 0.05 and ^∗∗^*p* < 0.01 vs. the SO group; ^#^*p* < 0.05 and ^##^*p* < 0.01 vs. the SAP group.

To confirm that ferroptosis was involved in SAP-induced intestinal barrier injury, we measured the iron content, MDA, GSH levels, and GPX4 activity in the ileal tissues. Compared with the SO group, the iron content in the ileum of the SAP group was significantly higher (Figure 2(a), *p* < 0.05), which was the key step in ferroptosis execution. Furthermore, the content of MDA in the ileal tissues increased significantly and the GSH levels and GPX4 activity decreased (Figures 2(b)–2(d), *p* < 0.01), which confirmed the severe lipid peroxidation in the intestinal tissues.

**Figure 3:**
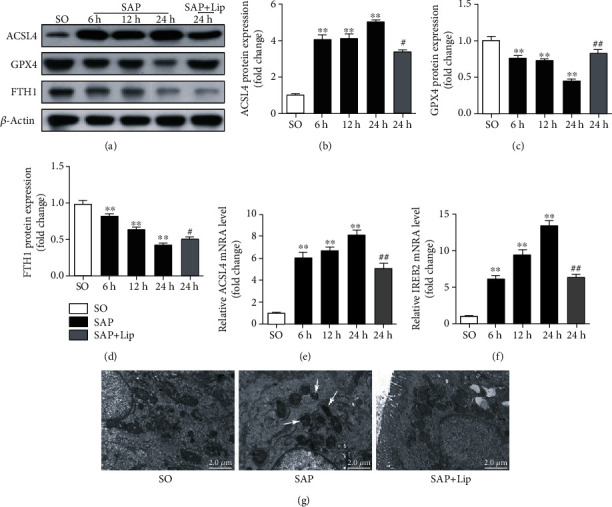
The expression of ferroptosis core proteins ACSL4, GPX4, and FTH1 in ileum was detected by Western blotting (a–d). Quantitative real-time PCR analysis of *ACSL4* and *IREB2* mRNA transcription (e, f). Using GAPDH as an internal control to standardize the data, the results showed folding change compared with the SO group. The ultrastructure of IECs was observed by TEM at 24 h (g). White arrows indicate shrunken mitochondria. All data are expressed as the mean ± SD (*n* = 10). The variables between groups were compared by Student's *t*-test and one-way ANOVA. ^∗^*p* < 0.05 and ^∗∗^*p* < 0.01 vs. the SO group; ^#^*p* < 0.05 and ^##^*p* < 0.01 vs. the SAP group.

We measured the expression of ferroptosis core proteins, ACSL4, GPX4, and FTH1, which are pivotal proteins in ferroptosis regulation [11, 12]. As shown in Figures 3(a)–3(d), the expression of the positive regulatory factor ACSL4 in the injured intestine was induced compared with that in the normal intestine (*p* < 0.01). At the same time, the levels of the negative regulatory factors, GPX4 and FTH1, were decreased (*p* < 0.01). In addition, we also found that the ferroptosis-related genes *ACSL4* and *IREB2* mRNA levels increased significantly in ileum after SAP (Figures 3(e) and 3(f), *p* < 0.01). These results showed that the difference between the 24 h SAP group and the control group was the most significant, so we speculated that ferroptosis may be more active at 24 h than at other times. Next, we observed shrunken mitochondria in the SAP group at 24 h by TEM (Figure 3(g)); this finding was consistent with the morphological characteristics of ferroptosis. These results indicate that SAP induces ferroptosis in injured intestines, suggesting that ferroptosis may be involved in SAP-induced intestinal barrier injury.

### 3.3. Ferroptosis Is Effectively Inhibited

Lip-1 is a specific and potent ferroptosis inhibitor, which has been shown to reduce intestinal ischemia-reperfusion injury [25]. It was administered to rats to determine the effect of ferroptosis inhibition on SAP-induced intestinal barrier injury. As shown in Figure 2(a), 24 h after the induction of SAP, Lip-1 significantly decreased the iron content in the ileum (*p* < 0.05). In addition, Lip-1 partially reduced lipid peroxidation, which was indicated by a decrease in MDA and increases in the GSH level and GPX4 activity (Figures 2(b)–2(d), *p* < 0.05). Furthermore, Lip-1 treatment effectively suppressed ACSL4 expression and rescued the expression of GPX4 (Figures 3(a)–3(c), *p* < 0.05). Lip-1 administration significantly decreased the expression of ferroptosis-related genes compared with the SAP group (Figures 3(e) and 3(f), *p* < 0.01). Furthermore, the morphological characteristics of the mitochondria in the SAP + Lip group were also significantly improved (Figure 3(g)). The changes in the above indexes indicate that application of Lip-1 effectively prevents lipid peroxidation and inhibits ferroptosis in the intestinal barrier injury of SAP.

### 3.4. Inhibition of Ferroptosis Attenuates SAP-Induced Intestinal Barrier Injury

In order to further confirm the existence of ferroptosis and its role in intestinal barrier damage induced by SAP and to determine whether inhibition of ferroptosis can attenuate intestinal injury, we observed the changes of serum biochemical indicators and histopathology after 24 h of SAP. According to the results shown in Figures 1(a)–1(d), treatment with Lip-1 significantly reduced serum AMY and LIPA activities and TNF-*α* and IL-6 levels compared with the SAP group (*p* < 0.01). Furthermore, serum DAO and endotoxin levels in the SAP + Lip group were significantly decreased (Figures 1(e) and 1(f), *p* < 0.01), thus indicating that the intestinal dysfunction was improved.

**Figure 4:**
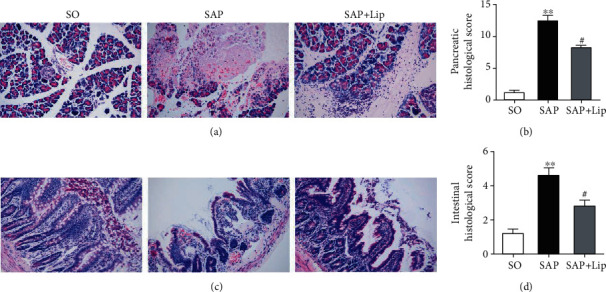
Effects of Lip-1 treatment on histopathological changes of pancreas and ileum in rats 24 h after SAP. Representative H&E-stained images of pancreas and ileum sections (original magnification, ×200). The pathological images of the pancreas (a). The pathological images of the intestine (c). Pathological scores of the pancreas and ileum (b, d). All values are expressed as the mean ± SD (*n* = 10). The variables between groups were compared by Student's *t*-test and one-way ANOVA. ^∗^*p* < 0.05 and ^∗∗^*p* < 0.01 vs. the SO group; ^#^*p* < 0.05 and ^##^*p* < 0.01 vs. the SAP group.

Histological changes in the pancreatic and intestinal mucosal tissues were evaluated by H&E staining 24 h after SAP. No morphological changes of pancreatic and intestinal mucosal injury were found in the SO group. Interstitial edema, acinar cell necrosis, and inflammatory cell infiltration were observed in the pancreas of the SAP group (Figure 4(a)). Compared with the SAP group, treatment with Lip-1 reduced pancreatic histological injuries and ameliorated histological scores (Figure 4(b), *p* < 0.05). As shown in Figure 4(c), there are representative signs of intestinal injury in the SAP group, including edema, bleeding, leukocytic infiltration, villous exfoliation, and mucosal cell necrosis. The intestinal tissues of SAP rats treated with Lip-1 had slighter histological changes and lower pathological scores than those of the SAP group (Figure 4(d), *p* < 0.05).

### 3.5. Inhibition of Ferroptosis Improves the TJ Protein Expression in IECs

**Figure 5:**
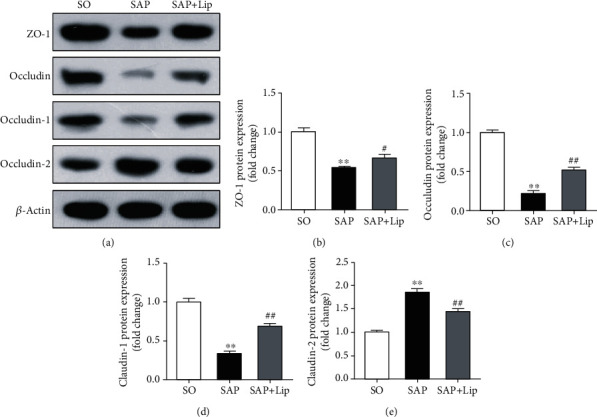
Western blot analysis of TJ proteins in ileum 24 h after SAP (a). Optical densitometry was used to determine the relative expression of ZO-1, occludin, claudin-1, and claudin-2 (b–e). Data are expressed as the mean ± SD (*n* = 10). The variables between groups were compared by Student's *t*-test and one-way ANOVA. ^∗^*p* < 0.05 and ^∗∗^*p* < 0.01 vs. the SO group; ^#^*p* < 0.05 and ^##^*p* < 0.01 vs. the SAP group.

TJ proteins play an important role in maintaining intestinal barrier function. In order to explore the possible relationship between ferroptosis and the expression of TJ proteins in SAP, TJ protein expression was detected by Western blot. The results showed that the expression of ZO-1, occludin, and claudin-1 in the SAP group was significantly lower than that in the SO group (Figure 5(a)–5(d), *p* < 0.01). Moreover, the expression of claudin-2 increased significantly compared to that in the SO group (Figures 5(a) and 5(e), *p* < 0.01). Lip-1 treatment significantly improved the expression of ZO-1, occludin, and claudin-1 (Figures 5(a)–5(d), *p* < 0.05), whereas claudin-2 expression was suppressed (Figures 5(a) and 5(e), *p* < 0.01).

**Figure 6:**
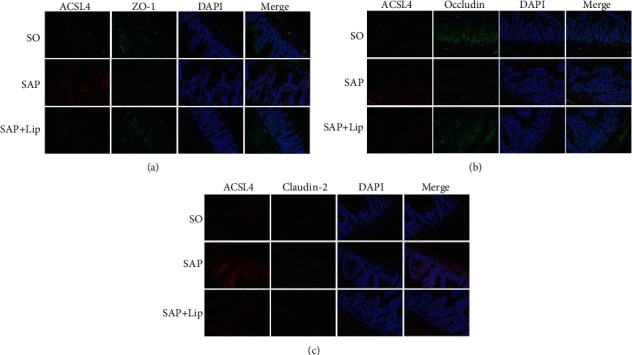
The expression of ACSL4 and TJ proteins in ileum was observed by confocal laser scanning microscopy. The intestinal sections of SO, SAP, and SAP + Lip groups were double stained with rabbit anti-ACSL4 (red), rabbit anti-ZO-1 (green), rabbit anti-occludin (green), and rabbit anti-claudin-2 (green). The nuclei of the cells were stained with DAPI (blue). (a) ACSL4 (red) and ZO-1 (green), (b) ACSL4 (red) and occludin (green), and (c) ACSL4 (red) and claudin-2 (green) (*n* = 10, each). Original magnification, ×200.

In order to determine the expression of ACSL4 and TJ proteins in the rat IECs, immunofluorescent double staining was performed. ACSL4 (red), ZO-1 (green), occludin (green), and claudin-2 (green) were observed at the junctions of IECs. As shown in Figure 6, there is a large amount of ACSL4 (red) accumulation in IECs in the SAP group, indicating that ferroptosis is activated in the intestinal epithelium. The concentration of ACSL4 in the SAP + Lip groups was significantly lower than that in the SAP groups. The granular staining of claudin-2 in IECs was the strongest in the SAP group but was almost undetectable in the SO group (Figure 6(c)). In contrast, the intestinal specimens of the SAP group showed only very weak ZO-1 and occludin in IECs (Figures 6(a) and 6(b)). However, ZO-1 and occludin levels were significantly improved after Lip-1 treatment compared to the SAP group. The differential distribution of TJ proteins indirectly reflected the degree of intestinal mucosal destruction. The results of the merged images were consistent with the Western blot results.

### 3.6. Inhibition of Ferroptosis Reduces Bacterial Translocation (BT)

16S rDNA-based sequencing was used to evaluate the existence of bacterial DNA in blood samples of SAP model rats. As shown in Table 1, bacteria were detected in thirteen peripheral blood specimens of the SAP group and the BT ratio was approximately 43.3%. However, bacteria were detected in the peripheral blood of only five specimens from rats treated with Lip-1 and the BT ratio was approximately 16.7%. The BT ratio in the SAP + Lip group was significantly lower than that in the SAP group (Table 2).

In each group, the 6 h group was labeled as A and 10 rats were numbered as A1, A2, A3,…,A10. The 12 h group was labeled as B, and 10 rats were numbered as B1, B2, B3,…,B10. The 24 h group was labeled as C, and 10 rats were numbered as C1, C2, C3,…,C10. The number of BT(+) rats is shown in the table.

### 3.7. Ferroptosis Inhibition Alleviates Acute Remote Organ Injury after SAP

**Figure 7:**
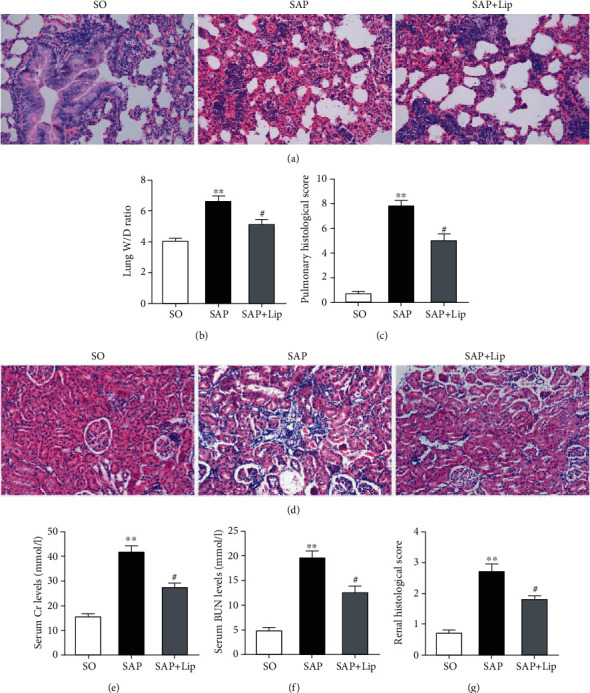
Lip-1 protects remote organs after SAP. Sections of remote organs (lung and kidney) were stained with H&E, and characteristic images were obtained under a microscope (a, d) (original magnification, ×200). The wet/dry ratio and pathological scores of the lungs (b, c). Serum Cr (e) and BUN (f) levels. Pathological scores of the kidney (g). All results were expressed as the mean ± SD (*n* = 10). The variables between groups were compared by Student's *t*-test and one-way ANOVA. ^∗^*p* < 0.05 and ^∗∗^*p* < 0.01 vs. the SO group; ^#^*p* < 0.05 and ^##^*p* < 0.01 vs. the SAP group.

Severe intestinal damage caused by SAP may lead to acute failure of remote organs such as the lung and kidney, which plays an important role in prognosis. The histopathological changes of the lung and kidney were observed 24 h after SAP. We found that inhibition of ferroptosis by Lip-1 alleviated histological injury and improved pathological scores of the lungs and kidneys (Figures 7(a), 7(c), 7(d), and 7(g)). Ferroptosis inhibition also significantly reduced lung edema (Figure 7(b)) and restored renal function (Figures 7(e) and 7(f)). These results indicated that inhibition of ferroptosis prevents SAP-induced lung and kidney injury.

Taken together, our results demonstrate the involvement of ferroptosis in SAP-induced intestinal barrier injury and reveal that Lip-1 protects against intestinal injury and reduces bacterial translocation by inhibiting ferroptosis.

## 4. Discussion

In the present study, we confirmed for the first time that iron- and reactive oxygen species-dependent ferroptosis is involved in IEC death in SAP-induced intestinal barrier injury. In addition, we observed that lipid peroxidation and intestinal barrier damage associated with SAP as well as bacterial translocation can be ameliorated by inhibiting ferroptosis.

SAP is a disease with rapid development and high mortality, which may be accompanied by multiple organ failure. During SAP, the integrity of intestinal epithelial barrier is destroyed, resulting in BT and a series of complications [8]. Recent studies have shown that initiation of lipid peroxidation and destruction of the dynamic balance of oxygen free radicals can lead to the ferroptosis of IECs [26]. Thus, we established a rat SAP model to explore the role of ferroptosis in SAP-induced intestinal barrier dysfunction. In this study, the changes of serum DAO and endotoxin and ileum morphology in SAP rats were observed, which indicated that intestinal barrier dysfunction and tissue damage were induced in the progression of SAP.

Ferroptosis is a new form of programmed and nonapoptotic cell death triggered by iron-dependent lipid peroxidation [27]. In our study, iron content and lipid peroxidation in the ileums of SAP rats were significantly increased, accompanied by a decrease in GPX4 activity. ACSL4 has recently been found to promote the esterification of arachidonoyl and adrenoyl into phosphatidylethanolamine (PE), which is an important process in ferroptosis execution [15, 28]. GPX4 facilitates the reduction of lipid peroxidation under the condition of ferroptosis, and many studies have shown that GPX4 is the main target of ferroptosis [13, 19, 29]. A recent study has shown that ferritin heavy chain 1 (FTH1) also plays an important role in ferroptosis and its abnormal expression leads to iron storage disorder and cell death by destroying the antioxidant defense function of cells [30]. In this study, the abnormal expression of typical markers including ACSL4, GPX4, and FTH1 and the upregulation of ferroptosis-related genes indicated that ferroptosis is activated in SAP-induced intestinal injury. In addition, we also observed the characteristic shrunken mitochondria of ferroptosis in IECs after SAP. These results suggest that ferroptosis is a cause of SAP-associated IEC death, which was further confirmed by administration of Lip-1, a specific ferroptosis inhibitor, to the rats.

The restoration or preservation of intestinal barrier function may reduce the incidence of SAP-mediated sepsis and may decrease the related mortality [31]. Largely, it is maintained by epithelial cells: IECs are joined by TJ proteins at their apical poles, forming a physical barrier [32]. Experimental and clinical studies showed that the systemic infection of SAP may be caused by the transmural migration of intestinal microorganisms [33]. Next, with the administration of Lip-1, we determined whether inhibition of ferroptosis could rescue intestinal damage caused by SAP. Our results indicated that Lip-1 partially alleviated lipid peroxidation, reduced the release of detrimental factors, ameliorated histological injury of intestine, and restored epithelial barrier function. More importantly, inhibition of ferroptosis significantly improved the expression of TJ proteins and reduced bacterial translocation. In addition, we also found that inhibition of ferroptosis alleviated the damage to distal organs in SAP, which is of great significance to the prognosis of SAP. Thus, we concluded that ferroptosis occurs in IECs after SAP, resulting in intestinal barrier injury; furthermore, the inhibition of ferroptosis improves this damage process.

Several ferroptosis inhibitors have been identified, including Fer-1 and Lip-1, which block pathological cell death in the kidney, brain, and other tissues [13]. In this study, the level of lipid peroxidation was attenuated after Lip-1 treatment in SAP animals and their ferroptosis and tissue damage were significantly improved. Therefore, we speculated that the tissue damage caused by ferroptosis was mediated by lipid peroxidation. Apoptosis [34], autophagy [35], and necroptosis [36] have been confirmed to be associated with SAP-induced intestinal barrier injury, but ferroptosis has yet to be reported. As a dynamic process, SAP-induced intestinal barrier injury involves complex mechanisms of cell death and IEC ferroptosis may only be a part of it. However, the complex orchestrated connections among these cell death processes require more in-depth research.

In conclusion, our study reveals that ferroptosis triggered by toxic lipid peroxidation is one of the mechanisms contributing to IEC death in SAP-induced intestinal barrier injury and inhibition of ferroptosis ameliorates intestinal mucosal injury and prevents bacterial translocation. These findings suggest that ferroptosis inhibition may be a new therapeutic method for SAP-associated intestinal barrier injury.

## Figures and Tables

**Table 1:** Bacteria species identified in BT(+) rat blood samples.

SAP group	Bacterial species	SAP + Lip group	Bacterial species
A3 (6 h)	*Escherichia coli*	A5 (6 h)	*Enterococcus aerogenes*
A4 (6 h)	*Enterococcus aerogenes*	B3 (12 h)	*Prevotella copri*
A8 (6 h)	*Streptococcus pneumonia*	B8 (12 h)	*Escherichia coli*
B2 (12 h)	*Escherichia coli*	C4 (24 h)	*Escherichia coli*
B5 (12 h)	*Enterococcus faecium*	C7 (24 h)	*Streptococcus pneumonia*
B8 (12 h)	*Citrobacter freundii*		
B9 (12 h)	*Streptococcus pneumonia*		
C1 (24 h)	*Prevotella copri*		
C4 (24 h)	*Enterococcus faecium*		
C5 (24 h)	*Enterococcus aerogenes*		
C7 (24 h)	*Prevotella copri*		
C9 (24 h)	*Escherichia coli*		
C10 (24 h)	*Citrobacter freundii*		

**Table 2:** Analysis of bacterial translocation in each group (*n* (%)). Categorical data were analyzed by chi-square tests.

Groups	*n*	BT(+)	BT(−)	*χ* ^2^	*p*
SAP	30	13(43.3)	17(56.7)	5.079	0.024
SAP + Lip	30	5(16.7)	25(83.3)		

## Data Availability

All data related to this paper may also be requested from the corresponding authors (email: phdzdl@yahoo.com).

## Abbreviations

SAP: Severe acute pancreatitis
IEC: Intestinal epithelial cell
BT: Bacterial translocation
TJ: Tight junctions
GSH: Glutathione
GPX4: Glutathione peroxidase 4
ACSL4: Acyl-CoA synthetase long chain family member 4
LPCAT3: Lysophosphatidylcholine acyltransferase 3.
Fer-1: Ferrostatin-1
Lip-1: Liproxstatin-1
AMY: Amylase
LIPA: Lipase
TNF-*α*: Tumor necrosis factor-*α*
IL-6: Interleukin-6
Cr: Creatinine
BUN: Blood urea nitrogen
DAO: Diamine oxidase
MDA: Malondialdehyde
TEM: Transmission electron microscopy
H&E: Hematoxylin and eosin.
